# Gender Equality in Diastasis Rectus Abdominis in Chronic Back Pain: A Model of M. Transversus Abdominis Motor Control Impairment

**DOI:** 10.3389/jaws.2024.12314

**Published:** 2024-05-01

**Authors:** J. P. van Wingerden, I. Ronchetti, G-J. Kleinrensink

**Affiliations:** ^1^ Spine and Joint Centre, Rotterdam, Netherlands; ^2^ Department of Neuroscience-Anatomy, Erasmus MC, Rotterdam, Netherlands

**Keywords:** diastasis recti, chronic pain, pregnancy, motor control, gender

## Abstract

**Introduction:** Diastasis rectus abdominis (DRA) is defined as an increased distance between the left and right muscle of the m. rectus abdominis. Pregnancy-related factors are assumed to be dominant factors in the occurrence of DRA. However DRA is not only found in peri-partum women but also in men and nulliparous women with back or pelvic pain. This study provides an inventory of the incidence of DRA in subjects with chronic back and pelvic pain. If DRA is common in both men and women then other factors besides pregnancy, like impaired motor control, should be explored as cause for DRA.

**Material and Methods:** This study was conducted with data from 849 back pain patients. Results from ultrasound assessment of the abdominal wall were combined with anamnestic data on age, gender, medical history and pregnancies (in women).

**Results:** There was no difference in Inter Rectus Distance cranial of the umbilicus (IRD above umbilicus) between men and women. Almost half of all women and men (45% and 43%, respectively) exhibit an increased IRD above umbilicus. The incidence of an increased IRD above umbilicus is twice as high in women below 30 years, compared to men below 30 years old. This difference is not observed for men and women above 30 years old.

**Discussion:** DRA occurs in women during pregnancy and increases with an increasing number of pregnancies. However, this condition does not affect significantly more women than men. Increased IRD above umbilicus already occurs in young men (mean age 30). Over 30 years of age, cranial of the umbilicus there is no difference in IRD between women and men. An alternative etiological mechanism is suggested.

## Introduction

**FIGURE 1:**
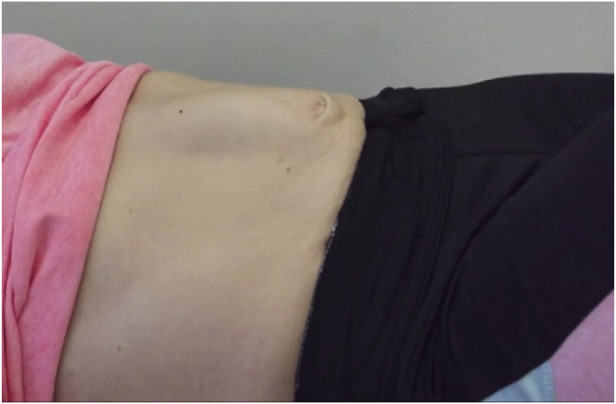
Typical example of pain patient showing bulging and diastasis while performing an active straight leg raise.

Diastasis rectus abdominis (DRA) is defined as an increased distance between the left and right muscle of the m. rectus abdominis. It is assumed that the inter-rectus distance (IRD) increases due to widening or stretching) of the linea alba [1]. In addition, when intra-abdominal pressure (IAP) increases, bulging of the abdominal content through the linea alba can occur (Figure 1). This bulging can vary from marginal to substantial, with both aesthetic and functional consequences. In literature, there is no consensus on conservative treatments for DRA [2, 3]. The results of surgical interventions for DRA also vary and hold significant risk for secondary incisional herniation [4, 5].

Conservative and operative interventions are performed despite uncertainty about the etiological mechanism behind DRA [6]. Since DRA is common in peripartum women, pregnancy-related factors have long been assumed to play a role in the development of DRA. [6], with a dominant notion being that pregnancy-related hormones weaken the linea alba, allowing it to stretch, consequently increasing the IRD.

The linea alba, the collagenous midline structure between both rectus muscles, is thick and strong, and its fibre structure is specifically fit for resisting lateral tensile force [7–10]. A recent study has shown that not only the linea alba but also the fascia (rectus sheath), that encloses the rectus muscles, in a transverse direction is very rigid [11]. Since the linea alba alone cannot be responsible for any potential increase in IRD, it is suggested that not just the linea alba but rather the abdominal wall as a whole stretches [12]. In addition, to this fact, DRA is found not only in peri-partum women but also in men and nulliparous women who suffer from back or pelvic pain [13].

It is assumed that DRA contributes to the development of back or pelvic pain [14–18]. However, there are indications that the aetiology is reversed: DRA does not cause back and pelvic pain; instead, low back or pelvic pain cause DRA. If this is the case, then a common denominator should be sought for the development of DRA both in pregnancy and in back and pelvic pain.

The purpose of this study is to make an inventory of the incidence of DRA in subjects with chronic back and pelvic pain. Based on common perceptions of DRA, it is hypothesised that the condition would be much more common in women in relation to pregnancy than in subjects with back and pelvic pain. If this is not the case, then the cause of DRA should no longer be sought in, for example, hormonal changes during pregnancy. Another etheological notion like a disturbed function of the trunk muscles is then more obvious. Such a disturbance in function has already been demonstrated in people with back or pelvic pain [19–22]. A question that subsequently arises is, to what extent can this disturbed or suppressed motor control be related to the development of DRA.

## Materials and Methods

In this retrospective study data were obtained from regular patient care, with written patient consent. Between 2019 and 2020, 849 patients were seen in a Dutch rehabilitation centre for the treatment of chronic back, pelvic or neck pain. Of those patients, 174 were excluded because they had predominantly neck problems (*n* = 29) or had incomplete data (*n* = 145). No other in- or exclusion criteria were applied. An ultrasound assessment of the abdominal wall was executed as an integral part of the diagnostic procedure. In addition, anamnestic data on the patients’ medical history and pregnancies (in women) was recorded.

By means of ultrasound examination, both the structure of the abdominal wall and the function of the abdominal muscles (m. rectus abdominis, m. obliques externus and internus and m. transversus abdominis) were assessed. Sonography has proven to be useful for such evaluation [23]. The IRD was assessed at 5 cm cranial of (IRD above umbilicus) and 5 cm caudal of (IRD below umbilicus) the umbilicus (categories: 0–2 cm, 2–4 cm or >4 cm). While patients lay in a supine position and slightly lifted their head and shoulders, the amount of bulging was visually assessed 0 = no bulging, 1 = slight bulging, 2 = moderate bulging or 3 = substantial bulging.

### Data Analysis

Data were analysed using Statgraphics Centurion version 18.1.16. The IRD above umbilicus and IRD below umbilicus and bulging were compared between selections from the population using the Kruskal-Wallis test. These selections were gender (male/female), age (≤30 years/>30 years) and pregnancy (0, 1, more than 1). Women who had never given birth and men were compared. The mean age of the group of nulliparous women was 30 years, and for comparison, the group of men was also selected with a mean age of 30 years. In addition, men with a mean age of 30 or younger were compared to men with a mean age older than 30. Furthermore, women with a mean age of 30 and younger were compared to women with a mean age over 30 years. The relationship between the degree of bulging and IRD was determined using the Spearmen rank correlation test, and *p* values less than 0.05 were considered significant.

## Results

Both male and female group had a normal distribution for age. No significant difference in age was observed between the male and female patient groups (Table 1). Overall, no difference in bulging or sonographically assessed IRD above umbilicus between men and women was found, although more women had an increased IRD below umbilicus. Notably 38% of both women and men, showed an IRD above umbilicus of 2–4 cm, leading to almost half of all women and men (45% and 43%, respectively) exhibiting an increased IRD above umbilicus (Table 2).

**TABLE 1:** Demographic data.

	Men	Woman all	Woman		
Pregnancies	n/a	n/a	0	1	>1
n	204	471	50	88	333
Mean age ±sd	43 ± 14	41 ± 14	30 ± 11	37 ± 10	45 ± 14
IRD Above umbilicus					
0–2	115	261	50	43	168
2–4	78	177	0	43	134
>4	11	33	0	2	31
IRD Below umbilicus					
0–2	199	420	50	76	294
2–4	5	47	0	12	35
>4	0	4	0	0	4

Note: The group of women was divided into nulliparous (0), uniparous (1) and multiparous (>1). Numbers are absolute values.

**TABLE 2:** Overall differences between males and females in bulging and in IRD above and below the umbilicus (all subjects).

	Bulging				IRD above umbilicus (cm)			IRD below umbilicus (cm)		
	no	slight	moderate	substantial	0–2	2–4	>4	0–2^a^	2–4^a^	>4
Male	80	10	7	3	56	38	5	98	2	0
Female	79	12	5	3	55	38	7	89	10	1

Notes: Values in %. Males, *n* = 204; Females, *n* = 471. There was no significant difference between males and females for bulging and IRD, above umbilicus. Below umbilicus, the difference was significant (Kruskal–Wallis test, *p* < 0.001), with a larger IRD.

^a^ Occurring more often in women than in men.

In the selection of nulliparous women (mean age 30 years), neither bulging nor increased IRD was found (Table 3). In the same age group of men however, bulging occurred in 9% of cases (7% slight, 2% moderate), and an increase in IRD above umbilicus occurred in 20% (18% 2–4 cm and 2% > 4 cm). These differences were all significant. Furthermore, IRD below umbilicus occurred in some men in this group (2%), but this was not significantly different from the nulliparous women.

**TABLE 3:** Overall differences in bulging and in IRD above and below the umbilicus between females (mean age 30 Years) without pregnancies (n = 50) and males in similar age group (n = 90).

	Bulging				IRD above umbilicus (cm)			IRD below umbilicus (cm)		
	no	slight^#^	moderate^#^	substantial	0–2	2–4^#^	>4^#^	0–2	2–4	>4
Male	91	7	2	0	80	18	2	98	2	0
Female	100	0	0	0	100	0	0	100	0	0

Notes: Values in %. There were significant differences between males and females for bulging and IRD, above umbilicus: (^#^) bulging and a larger IRD, occurred more often in men (*p* < 0.05 and *p* < 0.05). There was no significant difference in IRD, below the umbilicus between groups.

When the number of pregnancies was considered, a clear impact of pregnancy was visible (Table 4). Between the nulliparous and single parous women, there was a significant increase in visible bulging (11% slight and 2% strong), and this effect continued with additional pregnancies. Moreover, 27% of the population with more than one pregnancy exhibited increased bulging (14% slight, 9% moderate and 4% strong). The IRD above umbilicus also increased in more than half of women after their first pregnancy. While the incidence of IRD above umbilicus did not increase much after additional pregnancies (from 51% to 57%), the severity of the IRD increased. With additional pregnancies, the percentage of women with an IRD above umbilicus >4 cm rose to 12%. By contrast, the IRD below umbilicus increased after the first delivery from 0% to 14% and the distribution altered with additional deliveries (2–4 cm in 13% and more than 4 cm in 2%).

**TABLE 4:** Differences in bulging and in IRD above and below the umbilicus between nulliparous, uniparous and multiparous women.

	Bulging				IRD above umbilicus (cm)			IRD below umbilicus (cm)		
Pregn	no^#^	slight^#^	moderate^#^	substantial^#^	0–2^#^	2–4^#^	>4^#^	0–2^#^	2–4^#^	>4^#^
0	100	0	0	0	100	0	0	100	0	0
1	87	11	0	2	49	49	2	86	14	0
>1	73	14	9	4	43	45	12	85	13	2

Notes: Values in %. There was no significant increase from nulli- (*n* = 50) to uniparous (*n* = 88) women or from uni-to multiparous (*n* = 261) women. There was a significant increase in all parameters between nulli- and multiparous women (Kruskal–Wallis test, *p* < 0.001).

Since the population included women younger than 30 who had been pregnant, groups were also compared for age, independent of pregnancy, in Table 5. In difference with the results in Table 3, the results in Table 5 indicate bulging and an increase in IRD in women too, but no difference was found between women and men with a mean age of 30. After the men and women were divided into two groups: mean age 30 years vs. mean age >30 years, (not adjusted for pregnancies), the following results were observed. In both men and women, there was a significant increase in both bulging and IRD above umbilicus. While no difference in bulging was observed for men or women in the same age range, a difference in IRD above umbilicus existed between these groups, where women (37%, 2–4 cm and 3% > 4 cm) had an increased IRD above umbilicus twice as often as men (18%, 2–4 cm and 2% > 4 cm). For mean ages above 30 years old, there were still no differences in bulging between women and men. However, the group of men with an IRD above umbilicus of 2–4 cm was significantly larger than the group of women in the same age group. In all female groups, the IRD below umbilicus was more often larger than in men (9% and 12% in women and 2% and 3% in men).

**TABLE 5:** Overall differences in bulging and in IRD above and below the umbilicus between males and females.

	Bulging				IRD above umbilicus (cm)			IRD below umbilicus (cm)		
	no	slight	mod	subs	0–2	2–4	>4	0–2	2–4	>4
Men ≤30	91	7	2	0^a^ ^,^ ^b^ ^,^ ^c^	80	18	2^d^ ^,^ ^e^ ^,^ ^f^	98	2	0^g^ ^,^ ^h^
Women ≤30	86	10	3	1^b^ ^,^ ^c^	61	37	3^e^ ^,^ ^f^	91	9	0
Men >30	71	12	11	6^c^	38	54	8^f^	97	3	0^h^
Women >30	74	14	7	4	51	38	10	87	10	2

Notes: Values in %. Males: mean age ≤30, *n* = 90; mean age >30, n = 115. Females: mean age ≤30, *n* = 210; mean age >30, *n* = 261. *p*-value <0.05 are considered significant. Mod = moderate, subs = substantial.

^a^ Bulging is significantly different from woman ≤30.

^b^ Bulging is significantly different from men >30.

^c^ Bulging is significantly different from woman >30.

^d^ IRD, above umbilicus is significantly different from woman ≤30.

^e^ IRD, above umbilicus is significantly different from men >30.

^f^ IRD, above umbilicus is significantly different from woman >30.

^g^ IRD, below umbilicus is significantly different from woman ≤30.

^h^ IRD, below umbilicus is significantly different from woman >30.

Bulging was moderately, but significantly correlated with IRD above umbilicus but not with IRD below umbilicus. Moreover, IRD above umbilicus and IRD below umbilicus were also significantly correlated. Finally, the observed impairment of increasing IAP is related to increased bulging (Table 6).

**TABLE 6:** Correlations between bulging, IRD above and IRD below the umbilicus.

Variables	Correlation	*p*
Bulging vs. IRD Above	0.40	0.0000^#^
Bulging vs. IRD Below	0.29	0.2936
IRD Above vs. IRD Below	0.37	0.0000^#^

Notes: Sample size *n* = 675. *p* values < 0.05 are considered significant. (^#^).

## Discussion

The results of this study confirm the common assumption that DRA occurs in women during pregnancy. In the group of nulliparous women, neither bulging nor increased IRD was found (Table 3). However, after the first pregnancy, the incidence of bulging and increased IRD was far more pronounced (Table 4). It is interesting that bulging increases with an increasing number of pregnancies, while IRD above umbilicus and IRD below umbilicus increase predominantly in women who already suffer from this condition. However, there was no statistical difference between women and men.

In men (mean age 30 years) significantly more bulging and a larger IRD was observed when compared with nulliparous women (mean age 30 years).

This difference was not observed when these men were compared to all women with a mean age of 30 years. While bulging occurred to a similar extent, the increase in IRD was more pronounced in women than in men. This might indeed have lead to the assumption that pregnancy plays a role in the onset of DRA. However, based on the results in men above 30 years of age, some confusing observations were made.

Firstly, bulging is found to be similar in men and women in the same age group (Table 5). Secondly, an increase in IRD above umbilicus occurs more often in men than in women (62% vs. 49%).

Third, for IRD above umbilicus, there is no difference in the level of increased IRD, and IRD below umbilicus occurs only slightly more often in women than in men.

This analysis confirms the notion that pregnancy is a trigger for the onset of DRA. The question, however, is whether pregnancy itself is the aetiological factor. The finding that men in this study were similarly affected by DRA calls for reconsideration of the underlying mechanism for the emergence of DRA. The following question arises: could there be a general mechanism affecting both women during pregnancy and men. And even more interestingly: could there be a relation with suffering from low back and/or pelvic pain?

**FIGURE 2:**
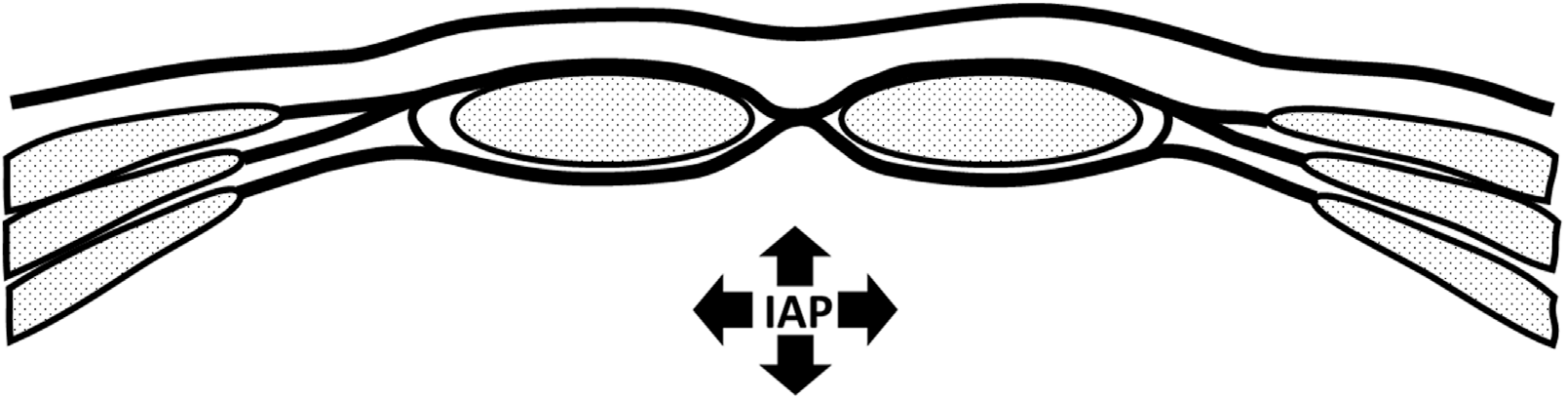
Schematic representation of abdominal wall. No contraction and low intra abdominal pressure.

Typical of this study population is that all subjects suffer long-lasting back or pelvic pain. For the past decades, much attention has been paid to the role and function of transversus abdominis (TrA) in relation to back and pelvic pain [24, 25]. TrA is involved in multiple tasks. Aside from providing stability to the spine TrA contributes to breathing and to regulating IAP [11, 22, 26]. In relation to IAP, a main task of TrA is to tension the posterior abdominal fascia sheath, including the dorsal rectus fascia and linea alba (Figure 2).

IAP is normally increased by co-contraction of the diaphragm, pelvic floor and abdominal wall [22, 26]. With impaired TrA function, as found in back or pelvic pain patients, contribution to the increase in IAP will be limited. Contraction of the diaphragm and pelvic floor will increase IAP to some extent. However, the abdominal wall will show bulging because TrA insufficiently tensions the posterior layer of the ventral abdominal wall (e.g., the posterior rectus fascia). The abdominal content will consequently be pressed outward between both rectus muscles, separating the muscle bellies and consequently increasing the inter rectus distance (IRD). Frequent repetition of this mechanism over an extended period of time may lead to structural separation of the bellies of the rectus abdominis, increased IRD, and elevated IAP. As a consequence, bulging in the area of the linea alba will then become visible.

A similar mechanism can be found in pregnant women. A growing embryo requires increasing abdominal space during pregnancy [27, 28]. It is suggested that this volume is created by stretching of the abdominal wall, especially the passive fascial tissue. Pregnancy-related hormones can increase the (temporal) slackening of connective tissue [29]. However, the question remains as to whether stretching of the passive tissues of the abdominal wall meets the spatial requirements and, moreover, whether the stretching of fascia tissue meets this spatial requirement at the right pace. An immediate way to create more space in the abdominal cavity is by relaxing the abdominal muscles, hence using the specific properties of muscle tissue [12]. Particularly TrA, due to its connection with the posterior rectus fascia and linea alba, will be included in this mechanism.

**FIGURE 3:**
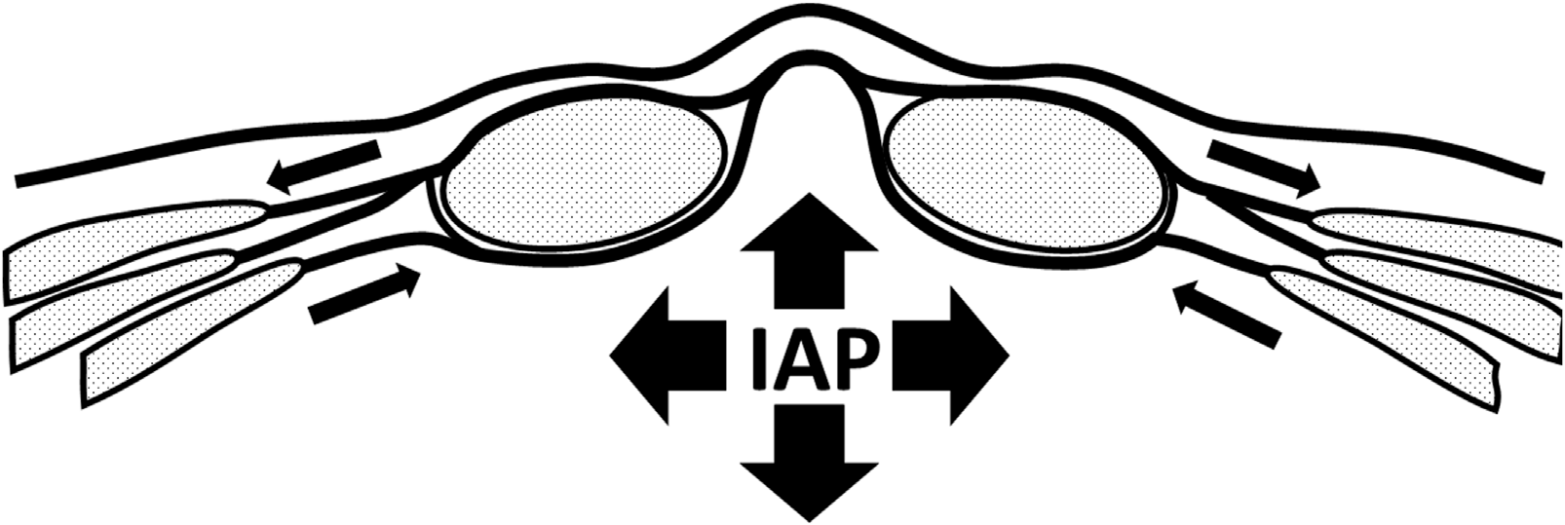
Abdominal with activation of rectus abdominis, for example, when performing a curl-up exercise. Intra abdominal pressure increases. Contraction of m. transversus abdominis is impaired, leading to bulging and increase on intra rectus distance.

Here, a common mechanism may be identified in: (a) back and pelvic pain and (b) pregnancy In both circumstances there might be neuromuscular suppression of TrA activity. And in both situations, the effect on abdominal wall behaviour is similar, leading to an increase in IRD (Figure 3).

In this study, no control group without low back or pelvic pain was included. Furthermore, the excluded group of neck patients was too small for analysis. Analysis over time of the occurrence of and increase in bulging and IRD may provide additional information on this mechanism. Additionally, despite the rather large cohort, the group size was still relatively small. It can be expected that some women in the group up to 30 years of age without pregnancy would exhibit bulging and an increased IRD. This was not found in the present study, probably due to the limited group size. In this study, only age, gender and pregnancy were included. Other factors, such as trauma (physical impact), congenital diastasis, BMI or abdominal surgery (including caesarean section) are confounding or even etiological factors for the occurrence of DRA. Regrettably these data could not be included in the present study. It is suggested that such factors are included in future studies.

Nevertheless, we have to be aware that surgical interventions aiming at solving DRA, may contribute to diminished TrA activation and consequently deterioration of abdominal wall function.

## Conclusion

The aim of this study was to make in inventory of the incidence of DRA in subjects with chronic back and pelvic pain. It is found that pregnancy plays a role in the occurrence of DRA in women. However, with increasing age, there is no difference in the occurrence of DRA in women or men with chronic back or pelvic pain. This leads to the assumption that not pregnancy itself but rather an underlying mechanism similar to pregnancy and low back and pelvic pain plays a role in the occurrence of DRA. It is postulated that suppression of activation of TrA may contribute to the onset and aggravation of DRA. From this perspective, conservative measures including restoration of motor control of the abdominal wall muscles (especially the transversus abdominis muscles) can be applied before considering surgical interventions for DRA.

## Data Availability

The raw data supporting the conclusion of this article will be made available by the authors, without undue reservation.
